# Explaining *Matching Michigan*: an ethnographic study of a patient safety program

**DOI:** 10.1186/1748-5908-8-70

**Published:** 2013-06-20

**Authors:** Mary Dixon-Woods, Myles Leslie, Carolyn Tarrant, Julian Bion

**Affiliations:** 1 Department of Health Sciences, University of Leicester, 28 Princess Road West, Leicester, LE1 6TP, UK; 2 Armstrong Institute for Patient Safety and Quality, Johns Hopkins University, Medical Institutions, 250 E Pratt Street - 15F, Baltimore, MD 21202, USA; 3 University of Birmingham, University Department Anaesthesia & Intensive Care Medicine, N5, Queen Elizabeth Hospital (old site), Edgbaston, Birmingham B15 2TH, UK

**Keywords:** Patient safety, Improvement programs, Context, Ethnography, Healthcare-acquired infections

## Abstract

**Background:**

Quality and safety improvement initiatives in healthcare often display two disconcerting effects. The first is a failure to outperform the secular trend. The second is the decline effect, where an initially promising intervention appears not to deliver equally successful results when attempts are made to replicate it in new settings. *Matching Michigan*, a patient safety program aimed at decreasing central line infections in over 200 intensive care units (ICUs) in England, may be an example of both. We aimed to explain why these apparent effects may have occurred.

**Methods:**

We conducted interviews with 98 staff and non-participant observation on 19 ICUs; 17 of these units were participating in *Matching Michigan*. We undertook further telephone interviews with 29 staff who attended program training events and we analyzed relevant documents.

**Results:**

One *Matching Michigan* unit transformed its practices and culture in response to the program; five boosted existing efforts, and 11 made little change. *Matching Michigan*’*s* impact may have been limited by features of program design and execution; it was not an exact replica of the original project. Outer and inner contexts strongly modified the program’s effects. The outer context included previous efforts to tackle central line infections superimposed on national infection control policies that were perceived by some as top-down and punitive. This undermined engagement in the program and made it difficult to persuade participants that the program was necessary. Individual ICUs’ histories and local context were also highly consequential: their past experience of quality improvement, the extent to which they were able to develop high quality data collection and feedback systems, and the success of local leaders in developing consensus and coalition all influenced the program’s impact on local practices.

**Conclusions:**

Improved implementation of procedural good practice may occur through many different routes, of which program participation is only one. The ‘phenotype’ of compliance may therefore arise through different ‘genotypes.’ When designing and delivering interventions to improve quality and safety, risks of decline effects and difficulties in demonstrating added value over the secular trend might be averted by improved understanding of program mechanisms and contexts of implementation.

## Background

Health systems worldwide face the frustration of a mass of evidence repeatedly showing problems in the quality and safety of patient care, but much less compelling evidence on how such problems can be tackled effectively [1-3]. The Michigan Keystone project [4] is one important exception. It was widely welcomed as a demonstration that improvement in patient safety could be secured through a large-scale interventional program, following its report of a dramatic reduction in rates of central venous catheter (central line) bloodstream infections (CVC-BSIs) in over 100 Michigan intensive care units (ICUs) [4]. The cohort study design used in evaluating the Keystone project could not establish a causal relationship between the program and the outcomes, but later research using controlled designs suggested that the effects were probably real. One retrospective analysis reported decreased in-hospital mortality in 95 of the Keystone hospitals compared with 364 control hospitals in the surrounding region [5], though declines in 30-day mortality were not significantly different in Keystone. A cluster randomised trial in a new non-Keystone setting found that hospitals using the program outperformed the secular trend towards decreasing infection rates [6].

Keystone’s success is sometimes attributed simply to the introduction of a checklist summarizing five evidence-based practices linked to infection control for CVCs [7]:

• Appropriate hand hygiene

• Use chlorhexidine for skin preparation

• Use full-barrier precautions during central venous catheter insertion

• Subclavian vein placement as the preferred site

• Review and remove unnecessary CVCs

Other analyses propose that a more complex set of mechanisms is more likely to explain the results, however [8]. These accounts recognize that the evidence-based practices are essential to any effort to reduce infection. They further recognize that checklists of these practices may have a potentially very useful role—not least by providing cognitive prompts or reminders of good practice. But a long history of failed or partial implementation makes it clear that evidence-based procedures and guidelines rarely directly impact on practice without facilitating mechanisms [9].

Based on a synthesis of the original program specification, the Keystone program team's experience, and social scientific expertise, a *post hoc* theorization proposed six mechanisms that might explain the effects seen in Keystone: generating isomorphic pressures (the desire to conform to group norms) among participants; creating a densely networked community, with strong links between units, encouraged by regular meetings and communication, that reinforced these norms and enabled sharing of information; taking on many of the characteristics of a social movement and reframing CVC-BSIs as a social problem capable of being solved through grassroots activism; using multiple interventions that functioned in different ways to shape of culture of commitment to doing better; harnessing data on infection rates, collected systematically, as a disciplinary force; and deploying mainly soft tactics, such as persuasion, but also making some limited use of harder tactics such as threatening sanctions against laggards [10].

**Figure 1:**
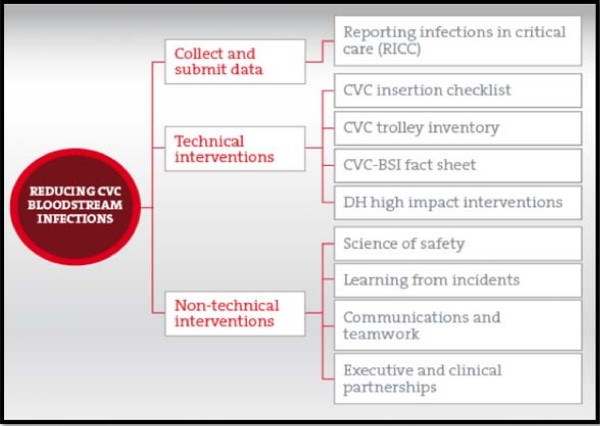
Matching Michigan.

Some have questioned whether the success of Keystone could be replicated under different circumstances [11]. An important opportunity to address this question was presented by *Matching Michigan* (Figure 1), a program led by the UK’s National Patient Safety Agency (NPSA) following the announcement in the National Health Service (NHS) Next Stage Review [12] of a ‘dedicated national patient safety initiative to tackle central line catheter-related bloodstream infections, drawing lessons from [the] remarkably successful Michigan initiative on the same topic’ (p45). *Matching Michigan* recruited more than 200 ICUs in England in four staged clusters. It ran between April 2009 and March 2011, with the results published in 2012 [13].

One interpretation of *Matching Michigan* is that it ‘worked’: it reported a final rate of 1.48 CVC-BSIs per 1,000 CVC patient days across 215 adult ICUs, thus ‘matching’ the rate of 1.4 seen by the end of the Keystone project [13]. However, the study design used to evaluate *Matching Michigan*—a modified form of a stepped wedge study involving clusters of ICUs that joined in staged sequence—permitted a more complex story to emerge, by detecting a strong secular trend towards decreasing rates of CVC-BSIs. Clusters of ICUs that were waiting to join the program were reducing their CVC-BSIs at the same rate as those already in the program, and infections acquired outside ICUs (in settings not targeted by the program) were reducing at the same rate as infections acquired inside ICUs (which were the target). This meant that the improvements seen in *Matching Michigan* could not confidently be attributed solely to program participation [13]. Thus, an alternative interpretation is that *Matching Michigan* did not work, in the sense that its additional impact on change that was occurring anyway was not clear.

The results of *Matching Michigan* illustrate two recurring themes in the literature on quality and safety in healthcare. First, many improvement programs fail to exceed the overall ‘rising tide’ [14-17], and thus have difficulty in proving that they have added value. Yet what has happened in non-intervention settings to secure improvement usually remains obscure in such programs, even though it is unlikely that the improvement is without cause. Second, *Matching Michigan* is a possible example of the so-called ‘decline effect’ associated with the challenges of replicating what appear to be initially promising results in scientific fields [18]. Neither secular trends nor decline effects have been well explored in the literature on quality and safety in healthcare. In this paper, using data from an ethnographic study conducted concurrently with the program, we seek to explain *Matching Michigan*’*s* apparently disappointing additional impact.

## Methods

We conducted an ethnographic study involving observations, interviews, and documentary analysis. Sites were purposively selected for participation in the study by sampling from the population of adult ICUs in England based on location, size, participation/non-participation in *Matching Michigan*, and types of patients served (speciality/general). ICUs joined the program in a series of four staged clusters (a design that allowed the secular trend to be detected). ICUs were sampled from each of the first three clusters; timing did not permit recruitment from the fourth. Research Ethics Committee approval for the study and research governance approval at each site was obtained.

Fieldwork involved non-participant observations on the ICUs. Face-to-face interviews were conducted with nurses and doctors of varying grades. These interviews were semi-structured, guided by a prompt list that had been developed through review of the literature, pilot work, and discussions in the project team. The prompt list was modified, albeit modestly, over the course of the project to explore some issues in more detail as their relevance became evident. The prompt list was not used rigidly and not all questions were asked of all participants, nor were they asked in the same order. This enabled flexibility and responsiveness to the particular interests, experiences, and roles of different participants. Further telephone interviews were conducted by JW (see acknowledgements) with staff, including managers and clinicians, who had attended *Matching Michigan* training events but were not part of the ethnographic study. Signed consent was obtained to interviews, which were recorded using a digital recorder, transcribed, and anonymized. In addition, we attended all training events and analyzed relevant documents relating to the program.

Analysis was based on the constant comparative method [19]. We initially generated open codes based on transcripts and fieldwork notes, which were then grouped into higher order organizing themes. Analysis was recursive, constantly moving from the specific to the more general, with the aim of producing more generalizable categories and explanations for our findings. This enabled us to identify commonalities and patterns across the large number of settings in which we conducted our study. We actively sought disconfirming cases to enable us to check our emerging constructs. The transcribed field notes and interviews were coded by ML using NVivo software, with checks on coding and interpretation undertaken by CT and MDW.

## Results

We conducted ethnographic visits to 17 of the 196 adult ICUs in England that participated in *Matching Michigan*. Most (96%) of ICUs in England took part in the program, but we were able to secure access to two non-participating units. Thus, 19 ICUs were included in the study in total. We conducted around 910 hours of observations and 98 semi-structured across the 19 ICUs. Our analysis is focused primarily, though not exclusively, on the 17 units that participated in the program. We carried out 29 telephone interviews with training event participants.

We found that *Matching Michigan* was challenged both in showing that it was outperforming the secular trend and in defending against the decline effect for reasons relating to the design and execution of the program, the national context into which it was introduced, the impact of individual ICUs’ histories, and local approaches to measurement and engagement. We found little evidence that *Matching Michigan* functioned in the way the post-hoc theory hypothesised that the original Keystone project had worked [10].

### Design and execution of the program

*Matching Michigan* was not an exact replica of Keystone across a range of dimensions (Table 1).

**Table 1:** **Selected differences and similarities between the Keystone project and *Matching Michigan***

**Keystone**	***Matching Michigan***
1. One cohort	1. Four cohorts (97% of English ICUs), including one pilot
2. Kicked off with 6 weeks of ‘immersion’ weekly teleconferences	2. Kicked off with data collection training
3/Whole-state workshops every six months—1.5 or 2 days (overnight), gradually becoming participant-led	3. Each cohort attended two ‘training events’ (0.5 or 1 day)—data collection and intervention
4. Continuous contact via teleconferences with 100~200	4. Teleconferences only at the beginning; discontinued after poor attendance. Webinars continued, but generally not well attended.
5. 5/6 months getting started with data collection & implementing the comprehensive unit-based safety program and daily goals; then Ventilator Acquired Pneumonia (VAP) and CVC-BSI interventions.	5. Initial period (3-6months according to cohort) of data collection only, then all interventions in any order. No VAP intervention.
6. Interactive web-based data entry tool allowing comparison with others	6. Interactive web-based data entry tool allowing comparison with others
7. Program team asked for infection rates to be reported by infection control practitioners independent of the ICUs.	7. ICUs allowed to determine method of data collection and reporting for themselves. Detailed definitions and guidance provided.
8. Targeted adult ICUs primarily	8. Targeted both adult and paediatric ICUs
9. Led by collaboration between prestigious out of state university and the state hospital association	9. Led by government agency

Some differences between the two programs were superficially minor; others were more far-reaching. The original Keystone program sought to drive clinicians’ use of five evidence-based practices through a combination of *technical* interventions (*e*.*g*., a checklist summarizing the five practices and a dedicated line insertion trolley/cart) and ‘*adaptive*’ interventions (*e*.*g*., the Comprehensive Unit-based Safety Program, or CUSP, intended to help in altering culture and behavior). *Matching Michigan*, in an example of a minor difference, used the term ‘non-technical’ to describe the interventions known as ‘adaptive’ in Keystone. These non-technical interventions were not labelled as the CUSP (the term used in Keystone), but their content was largely the same as the original.

The content of the technical interventions used in *Matching Michigan* was largely similar to Keystone. What was much more different was the provenance and freshness of these technical interventions for the target audience. Where Keystone had introduced a set of five evidence-based procedures summarized into a checklist that was new to participating units, *Matching Michigan*’*s* procedures were based on two pre-existing Department of Health CVC ‘bundles’ of procedures known as ‘High Impact Interventions.’ The two bundles (one for insertion and one for ongoing care) had already been established policy since 2007, two years before the program launched. Thus, apart from an updating of the insertion bundle to recommend that hats and masks be worn during CVC insertion, the evidence-based procedures were not new in England at the launch of the program, and they were closely associated with established central government policy. English ICUs had already had significant exposure to the evidence on procedural good practice for insertion and care of catheters by the time the program launched; the bundles had been heavily promoted by the Department of Health and by other agencies over a significant period.

The organization of Keystone and *Matching Michigan* differed significantly, though in ways that might not be evident from a quick examination of the program components. Both programs held meetings for staff from participating ICUs. However, in contrast to Keystone’s model of initial immersion coaching and six-monthly residential workshops, with all participating units meeting at the same time, *Matching Michigan* offered two non-residential training events for each of the four clusters as they entered the program. The first training event for each cluster covered data definitions and data collection and the second the program’s interventions. These training events were described by participants as professionally organized and effective in stimulating interest. Participants saw the opportunity to hear from peers as especially useful both for leadership and for practical learning about how to improve practice:

‘I thought it was really well organized and quite inspirational really, you know, there’s a big culture thing to consider when you’re making quality improvements, and that is, if another consultant [attending physician] has done it, and is signed up to it, that has huge impact.’ (Senior nurse, participant 25)

The training events did, however, encounter some challenges. Some ICUs sent large, eager teams to the training events; others sent one or two individuals, who had sometimes been unwillingly volunteered. Participants understood that they were being asked to collect data and ensure the CVC care bundles were fully implemented, but some appeared to have difficulty in understanding what they were required to do for the non-technical elements of the program. The sessions were inclined to become bogged down in distracting criticisms of the definitions or anxieties about the effort required to collect the data:

‘[The first training session] featured a lot of sniping between consultant microbiologists about how the [program] actually defines associated and [related] CVC-BSIs.’ (Infection control nurse, participant 11)

Participants from different ICUs did not meet again after the training sessions, and efforts to engage them in teleconferences and webinars suffered from low participation rates. This meant that the ICUs involved in *Matching Michigan* did not have the experience of being part of a collaborative community working together towards shared goals theorized to have occurred in Keystone.

### National context

At *Matching Michigan* training events, lack of clarity about how well or how consistently the recommended practices were being implemented was presented as a rationale for the program. It was suggested to participants that the program would, through its interventions, promote adherence to the procedures specified in the Department of Health bundles and have a more general impact on patient safety. *Matching Michigan* was promoted by the organizers as a clinically led, cooperative program, and participants were assured that their infection data would be used only for learning. However, the national context into which *Matching Michigan* was introduced meant that the program faced some scepticism. In contrast to Keystone, a centrally-led emphasis on infection control had been a feature of the English NHS since the mid-2000s (Table 2).

**Table 2:** History of infection control efforts relevant to central venous catheters

2001	Mandatory reporting to the Health Protection Agency (HPA) of MRSA bacteraemia.
2003	Report of the Chief Medical Officer: Winning ways: guidance to reduce healthcare associated infection in England.
2004	Mandatory reporting of *Clostridium difficile* infection (HPA website) 2004 Hospital in Europe Link for Infection Control through Surveillance of Nosocomial Infections in ICUs protocol. http://helics.univ-lyon1.fr/helicshome.htm
2005	DoH Saving Lives program—NHS High Impact Interventions (NHS-HII), modelled on Institute for Healthcare Improvement bundles.
2006	Health Act 2006: Department of Health Code of Practice gives new powers of inspection to the Healthcare Commission. Superseded by the Health & Social Care Act 2008
2008	2008 Health and Social Care Act 2008: required registration with the Care Quality Commission: duty to protect patients against HCAIs. New code of practice.
	http://www.dh.gov.uk/en/Publicationsandstatistics/Publications/PublicationsPolicyAndGuidance/DH_081927
2008	Patient Safety First sponsored by National Patient Safety Agency (NPSA), NHS HII, and Health Foundation, includes interventions to reduce CVC-BSIs http://www.patientsafetyfirst.nhs.uk/content.aspx?path
2009	Some NHS trusts participated in CQUIN (Commissioning for Quality and Innovation) schemes that made a percentage of their incomes dependent on demonstrating compliance
2011	Mandatory reporting of MRSA and *Escherichia coli* bacteraemia.

These government-led efforts were widely seen by staff as harsh and coercive. *Matching Michigan* was perceived by some as just the latest in a long parade of similar top-down initiatives:

‘There’s a huge amount about [the infection control agenda] that’s dogmatic … with the consequences of not hitting the targets being so dire [and] unforgiving central policies … And I think a lot of it has been driven like that.’ (Consultant, participant 20)

Those who saw *Matching Michigan* simply as another externally mandated program had little inclination towards genuine engagement. A multi-disciplinary team was appointed to lead the program, including a senior ICU physician who had previously been president of the European Society of Intensive Care Medicine (JB, one of the authors of this article), and a senior clinical intensive care nurse who had been head of the UK Critical Care Nurses’ Association. Despite this, the location of the program in a government agency rather than a professional organization or research collaboration appeared to contribute to an alienating sense of ‘distance’ on the part of some front-line clinicians: contrary to what was theorized to have occurred in Keystone, *Matching Michigan* was often seen as imposed from outside and lacking in professional ownership. Some participants remained suspicious about the potential for the data to be used for performance management or public shaming purposes:

*‘Because the NPSA is a Department of Health arm’s length body, there is certainly a view by a lot of clinical staff that oh, if it’s coming down from there then, you know what that is all about. So I think there is certainly a limitation attached to that*.*’ (Senior Nurse, participant 104)*

‘[*What was needed was] a consultation so that it feels like what we are implementing is coming from within, first of all…If we had taken what Matching Michigan had done, not called it Matching Michigan but taken the same things and then applied it as something that came through for example the Intensive Care Society or [another professional society]… it was dumped on top of us from above and we had no option in it.’ (Consultant, participant 3)*

### Variability in ICUs’ responses to the program

We found considerable variability across individual ICUs in their responses to *Matching Michigan*’*s* calls for data and implementation of the technical and non-technical interventions it recommended. Interviews and ethnographic observations suggested that local, unit-level responses to the program could be largely distinguished not by the degree of compliance with the program’s requirements, but by the extent to which staff in units attributed their behavior and practices to *Matching Michigan*. We identified three characteristic responses:

1 Transformed (one unit) where the program was seen by staff as having produced radical improvement in care.

2 Boosted (five units), where the program was credited with having reinforced existing good practice or supported further improvements.

3 Low Impact (11 units), where staff attributed little of their behavior and practices to the program, instead seeing the influences on what they did as coming from elsewhere.

We did not find a relationship between which cluster ICUs joined and their response to the program, but our study design does not fully exclude this as a possibility. Despite the variability we observed, most staff from across the ICUs believed that both their practices and their responses to the program were ‘normal’ and typical of all. This is likely to have occurred because of limited contact between program participants.

#### *Collecting data on infections*

All 17 ICUs in the ethnographic study that were participating in *Matching Michigan* developed systems for collecting data on CVC bloodstream infection rates, and all but one reported their rates to the program’s central database for at least some months of the program. On 16 of the participating units, staff attributed the introduction of a data collection system to *Matching Michigan*; the exception was a unit that had begun collecting and feeding back data as part of their involvement in an earlier initiative. For the 11 Low Impact units, establishing the data collection system was the most prominent, or only, feature of their response to the program.

#### *Technical interventions*

Because our study was not an audit, and we did not use a structured tool to assess practices, we did not produce precise estimates of compliance with *Matching Michigan*’*s* technical standards. Nonetheless, our observations suggested that the evidence-based practices summarized in the CVC care bundles, and the technical interventions to support them, were being implemented across all of the units in our study. On all units where we conducted observations (including the two not participating in *Matching Michigan*), the care bundles were known to staff and were widely used. Hygiene practices during insertions were mainly very good: chlorhexidine was routinely used to prepare patients’ skin, and handwashing was consistently good. Most ICUs were using a dedicated central line cart and/or a pack; most, too, were using full barrier drapes. Most were aware of the need to monitor CVCs and to remove them as soon as possible, though there was evidence that removal practices varied by individual physician. However, use of a checklist of good practice was variable: only eight of the 17 participating ICUs recognized that a checklist was intended to be used concurrently with CVC insertion (rather than as *post hoc* audit tools), and only two were fully consistent in using a checklist in this way.

It was usually not possible to identify *Matching Michigan* as directly influencing technical practices and behaviors; indeed, on any given unit it was difficult to isolate the effects on practices of any single program, initiative or intervention. Instead, most participants gave accounts of an incremental history of improving technical practice. The main exception to this was the Transformed ICU. This unit demonstrated a high level of consistency in compliance with the technical interventions, and staff in this unit explicitly and confidently attributed recent improvements to their participation in *Matching Michigan*. The five Boosted ICUs also demonstrated high (though not always perfect) reliability in applying the technical interventions. They credited the program with helping them sustain or enhance improvements already made, but did not identify it as the only influence on their behavior in relation to CVC care. In the 11 Low-Impact ICUs and in the two non-participating units, staff did not identify the program as having any influence on their behaviors and practices. In some of these units, compliance was generally high, but was less consistently good on others, and in some units appeared to be strongly influenced by which senior staff were on duty:

[Consultant] said, ‘we just have to be vigilant about thinking about how long the lines have been in.… He is careful to check patients’ [central lines]. It wasn’t something I necessarily saw with the other consultants. I didn’t see that they were very explicitly vigilant about how long the central lines were in,’ (Fieldnotes)

Innovations such as bio-patches, specially coated catheters, and new techniques for aseptic practice were introduced by many units during the period of our observations. On the whole, staff did not attribute these new developments to *Matching Michigan*, although staff on some of the Boosted and Transformed units described using the program strategically to implement the changes. Telephone interviews suggested similar behaviors:

‘What I am doing is focusing on our own needs though the project. Matching Michigan was merely a hook because it enabled us to do all the things that we wanted to do.’ (Senior nurse, participant 119)

#### *Non-technical interventions*

Participants’ understanding of *Matching Michigan’s* non-technical interventions (Figure 1), intended to change culture and behavior, was generally weak. ICUs were, as they had been in Keystone, asked to assemble a local *Matching Michigan* safety team including nurses, doctors, and senior executives to provide leadership and coordination for the program. On the Transformed and Boosted ICUs, the safety team generally functioned well. On the remaining ICUs, it existed in name only or did not function optimally. In one ICU, it only ever met by email; in many others, its main function was the production of infection data. The safety teams varied in the commitment and enthusiasm they invested in the program. They were often unsure of what the non-technical interventions required them to do, were sceptical of the benefits of the interventions, or did not ensure that the interventions were implemented. Most (11) struggled to involve executives.

Safety Surveys—one of the non-technical interventions intended to provoke local discussion and reflection—were distributed in only three of the 17 ICUs. In very few ICUs was there evidence that ‘learning from one defect a month’ was introduced as a result of *Matching Michigan*, nor was formalised or standardised practice in relation to daily goal-setting newly implemented. Only three ICUs publicly displayed CVC-BSI data so that staff were aware of their own unit’s rate. Strongly hierarchical and sometimes negative safety cultures persisted on some ICUs:

‘Working with different consultants is that I would say probably without exception as registrars [residents] we do what the consultant tells us to do, certainly where they’ve got very strong personalities, so I’ve put central lines in with just a pair of gloves with the individual who does it like that …because that’s how he does it, he won't let you do any other way and as registrars you're under pressure to do things as the consultant wants you to do them …[or] you know makes the rest of your day miserable.’ (Junior doctor, participant 30)

### Influences on variability in responses to the program

Our analysis suggested that much of the units’ variability in response to *Matching Michigan* could be explained by variability in infection rates and measurement, local histories, and local leadership.

#### *Infection rates and measurement*

Program organizers emphasized at training events that the impact of previous efforts to control CVC-BSIs was unknown, given the absence of a national infection data collection system. They also emphasized that unless ICUs knew their own rates they could not be sure that they were providing safe care. It was anticipated that discovery of high rates of infection would stimulate change:

‘What we did not have in this country was a measure of the outcome.... Key stakeholders in the country [were saying to us], ‘well we are fine in England, we are already matching Michigan. We have had the technical interventions. We have got it all sorted in England. So go away.’ And I actually did say to them, ‘Well, could you tell me what your rates of infection are?’ And they said ‘Oh I don’t know.” (Matching Michigan program team member)

The starting rate for the program turned out to be quite different from Keystone, where the initial mean rate was 7.7 BSIs per 1000 catheter days. The first of the four clusters of adult ICUs in *Matching Michigan* reported an initial mean rate of 3.7 BSI per 1000 catheter days. Though this was less than half the initial rate in Keystone, it did suggest room for improvement. However, each of the three successive clusters joined the program on the trend line, with an initial infection rate similar to the post-intervention level of the preceding cluster. This indicated that substantial improvement occurred outside the program throughout the period it was running. In any given month, almost two-thirds of units across the program were reporting no infections.

In the Transformed and the Boosted ICUs, collecting infection rate data had a generally positive impact on practice. These units established relatively robust data collection systems [20] and the data were generally accepted by staff locally as credible. Discovery of a low infection rate did not necessarily undermine the program’s aims; low rates on these units were used to celebrate and reinforce good practice. Discovering previously unrecognized high rates of CVC-BSIs, on the other hand, did have the intended program effect of driving change on these units:

‘If I’m honest right before we started, we didn’t think we were that bad.… We thought, you know, [we] don’t really have a problem with central line infections. But I think what it was, nobody ever looked to see whether we were any good … and when we compared our infection rates, actually they were far worse than any of us ever realized.’ (Senior nurse, participant 43)

‘It was hard to sit and have the error of your ways pointed out when you actually already knew [how things ought to be done]. So, I think enough of us felt like that we put the wheels in motion really.’ (Consultant, participant 42)

However, *Matching Michigan* data on infection rates could have effects that were in the opposite direction from those intended. Low-impact units varied substantially in the extent to which they established robust local data collection systems and how far the data were regarded as credible by clinicians locally. On some units, evidence of high infection rates was dismissed as poor quality data, and change was thus stymied. On some others, low infection rates were taken as evidence that no change was needed—but the data were not always collected accurately enough to justify such a conclusion. This meant that sometimes very hierarchical cultures that were not fully supportive of patient safety were reinforced, and that opportunities for improvements in practice went unrecognized.

#### *Local histories*

Local histories of efforts to control CVC-BSIs were deeply implicated in the differing responses to the program. In the Transformed unit, previous attempts to improve practice had been largely ineffectual. The program was seen by staff locally has having provided the tools and techniques they needed to make change, where nothing had seemed to work before:

[Consultant says] ‘Matching Michigan has genuinely made a real difference here … We’ve seen some really quite big changes around here … you can see real actual evidence of improvement in patient outcomes.’ (Fieldnotes)

On the five Boosted units, the program was absorbed as part of a local narrative of cumulative improvement. Staff on these units described having already recognized, long before *Matching Michigan*, that central line infections were a problem that they needed to tackle. These units had made significant gains before *Matching Michigan*, but recognized that there was still some room for further improvement. On these units, local leaders were keen to drive improvement, but had sometimes experienced barriers or resistance to change. These individuals explained how the program helped them consolidate their gains or make new improvements. Having a national, centrally led program was helpful because it enabled them to ask for resources, demonstrate that proposed changes were evidence based, persuade reluctant colleagues that conformity with good practice was now compulsory, and learn how their infection rates compared with other units:

‘We already had things like a lines trolley in place and I think that worked reasonably well. Perhaps wasn’t always stocked as well as it should be.... Being involved in Matching Michigan project certainly sort of tightened up vigilance.… We certainly didn’t have an insertion checklist, and this certainly wasn’t the sort of the culture whereby everybody felt that line infections were pretty much preventable. [The change was] the continuous measurement system, … actually doing the checklist and publishing the results that we were getting back.’ (Consultant, participant 114)

In the Low-Impact units, a few staff were resentful or hostile to the program, but more commonly they expressed apathy, exasperation, and bewilderment. On these units, staff argued that they had already invested heavily in changing practices to reduce CVC-BSIs in response to previous policy initiatives, that the prevalence of CVC-BSIs had already greatly diminished, and that the program was a largely superfluous data collection exercise. ICU staff on the Low-Impact units were often unsure what was new or distinctive about *Matching Michigan*, given that the technical practices were the same as those that had already been adopted as policy. Many saw it as addressing a problem that they believed had already been solved:

‘Compared to what we were already doing it seemed to be that wearing a hat [was the only difference] basically. We were already going over to packs anyway.’ (Consultant, participant 110)

Staff reported that they could not understand why CVC-BSIs in ICUs were being targeted by a dedicated program, when what they saw as other, more pressing problems—such as ventilator-acquired pneumonia or CVCs in non-ICU settings—were being neglected. Some saw the program as a failure to respect what had already been achieved by ICUs. Even its title caused resentment: in one unit, a file about the program was defaced, with ‘*Matching Michigan*’ scribbled out and ‘Exceeding Michigan’ written in:

‘There is sort of an attitude that, actually, it’s come in a little too late.’ (Senior nurse, participant 88)

What was notable about these Low Impact units was that these views were held not only by the staff ‘on the ground,’ but often also by those on the safety team set up locally to implement the program. These local leads were not always fully convinced of the need for, or value of, Matching Michigan.

#### *Leading the program into practice: the importance of creating local coalition and consensus*

Perhaps the single most important influence on program response by individual units—either in promoting or resisting change—was the extent of consensus and coalition among the senior medical and nursing staff on individual ICUs. The commitment, characteristics, and skills of local leads were pivotal. Transforming or boosting of efforts was most likely to occur when those locally charged with implementation were sincere in their beliefs about the value of the program, were able to create transdisciplinary alliances, had local credibility among peers, were prepared to tolerate debate but exercise firmness, and used multiple tactics including role modelling, persuasion, sanctioning, reminders, and constant feedback:

‘Cultural change is the biggest threat, because all of a sudden it fundamentally means that what you’ve been doing so far is maybe wrong, or people don’t value what you’ve done for the last 15 years. People think ‘oh, there’s the smartarse telling us how we’re supposed to do things.’ So there were a lot of discussions and persuasion.’ (Consultant, participant 106)

[Consultant says] ‘I think it’s been successful because it’s a unifying program, it’s one of the few things that we’ve done that hasn’t been just a doctor thing, or just a nurse thing, it’s involved the doctors and the nurses together.’ (Fieldnotes)

Authoritative and unwavering support from senior consultants was especially important in enabling nurses to act as a disciplinary force for junior doctors, who performed most CVC insertions:

‘So the fact that the lead consultant was passionate about it helped us to bring about change. [We could] actually [say to the junior doctors,] ‘You will gown up! You will put a mask on!’ And that was coming from [the lead consultant] as much as it was from [the nurses].’ (ICU Outreach Nurse, participant 111)

In the transformed unit, collecting data for the program revealed previously unrecognized high rates of CVC-BSIs. This shocked unit staff into action; a local leadership team emerged who used the program’s tools and techniques to secure change, and collecting data over time confirmed that the interventions were effective in reducing rates to zero from an initial high rate. *Matching Michigan,* staff in this unit reported, was the first that came from and was owned by the ICU, rather than being imposed from the outside. Led by a young and determined entrepreneurial consultant, a team was developed that crossed intra-professional hierarchies and inter-professional work domains. This individual and his consultant colleagues went about rebuilding the unit culture according to the program’s goals. He described how he and his colleagues modelled Matching Michigan’s preferred practice for the insertion of CVCs and insisted on compliance from junior doctors. Both junior and senior medical trainees, as well as nurses were included in the data collection process. He used the introduction of the checklist and observation as ways of flattening unit hierarchies and empowering nurses and junior medics to act on any breaches of aseptic technique they observed:

In the very beginning we made sure that it was the charge nurse, or one of the sisters, who would take the role of the observer. So [we chose someone who would] feel more confident, and was actively encouraged to interrupt if there was something [wrong with our technique]…[We did this] so that the juniors could see. And if one of the consultants was putting the central line in we would make sure that [a nurse observed] us as well. To make sure that people see it applies for us exactly as for anybody else…it’s the same rules apply for everybody. If we put a line in or [one of the junior medics does], it doesn’t matter.’ (Consultant)

In the units where *Matching Michigan* had less impact, senior consultants were not persuaded that all elements of the program were grounded in high quality evidence, saw it as an illegitimate policy or bureaucratic intrusion into professional work, or deemed it irrelevant to their concerns and interests. Apparently minor issues—such as the recommendation to wear hats and masks during CVC insertion—caused irritation and resistance in some settings because they were perceived to lack sound evidence. If local program leads did not successfully build consensus among senior staff that central line infections were a problem, and that *Matching Michigan* was the right answer to the problem, the program was inclined to stall:

‘I recall from reading that there wasn’t anything that made me change my practice. I haven’t seen any people [using hats and masks] and I certainly wouldn’t you know ask my juniors to do that.’ (Consultant, participant 87)

‘It can be a struggle. It can even be difficult if you’ve got one [consultant] who’s a real advocate and saying, ‘Yeah this is the best thing since sliced bread!’ And then you’ve the six others who are like, ‘What a load of crap, we’re not using it.” (Nurse, participant 91)

Executive involvement that generated enthusiasm, conferred authority, and allocated resources was helpful to the implementation of the program in local settings. Where executive involvement was limited to exhorting the ICUs to produce ‘the numbers [of infections],’ it reinforced a view of the program as a mandated, performance management national audit, and risked undermining the program’s aims:

‘They were getting e-mails [from the hospital executive] saying, ‘Why haven’t you submitted your data? So you go back to just tick boxing.… There’s a complete lack of interest now in line insertion.’ (Senior nurse, participant 40)

## Discussion

Our qualitative study is consistent with the quantitative evaluation of *Matching Michigan*[13] in suggesting that improvements in infection control practice for CVCs preceded the program and were likely to have continued to occur thereafter, but could not easily be directly attributed to the program in many instances. Consistent application of the evidence-based procedures for controlling infection risk in insertion and care of CVCs were likely to be the single most important determinant of rates of CVC-BSIs. There is no serious question about whether these practices ‘work,’ but our study makes it clear that there were multiple routes to promoting reliability in their implementation. English ICUs had a much lower starting rate of infection than in Keystone, suggesting that they had already made gains in infection reduction without the program, and improvements continued to occur in units in clusters that were waiting to join the program over its course. Compliance with good practice and low CVC-BSI rates could therefore be achieved by units without participating in *Matching Michigan*. Compliance with procedural good practice and low infection rates could also be achieved by units in the program without implementing—or only partially implementing—the program’s non-technical interventions. The phenotype of compliance with good practice could therefore arise in different ways, of which full and authentic engagement in the program was only one. That improved compliance could occur through many different routes or ‘genotypes’ may help to explain the finding that *Matching Michigan* did not exceed the secular trend; most ICUs had begun incrementally improving their practices before the program began, and continued to do so thereafter, sometimes introducing innovations not part of the program. Our analysis suggests that whatever explains the declines in central line infections observed over the course of *Matching Michigan*, it was not the set of mechanisms that were theorized [10] to have explained the effects seen in Keystone original.

The program bore a strong resemblance to the original, but primarily at the level of components rather than hypothesized mechanisms [10]. There was little sense that *Matching Michigan* built the social movement thought to have characterised Keystone, not least because there was so little contact between participants over the course of the program. Absence of ongoing contact also suppressed the possibility for the remaking of professional norms; participants believed that whatever they were doing locally was standard. The delivery vehicle mattered hugely. Keystone may have been able to engage emotional commitment and create a sense of a community-based enterprise by mobilizing the star qualities of its leaders, by building on the bonds people felt with their local hospital association, and by partnering with a highly prestigious out-of-state university with a track record of achievement in the area of patient safety [11]. ICU staff in England did not feel the same affection, identification with, and sense of ownership for a program led by a government agency across four cohorts, notwithstanding the clinical leadership provided by a senior ICU physician and nurse. Given that professionals prefer to take their directions for performance from inside rather than outside the clinical community [21], this was not a trivial problem. These features may help to explain the possible decline effect between the original Keystone program and the attempt to replicate it in England. They suggest that program fidelity needs to be assessed holistically, with a focus on mechanisms of change as well as program components [10].

The challenges of program design and delivery were intensified by the contexts into which *Matching Michigan* was introduced. Staff’s experience of previous centrally-led programs as harsh and performance-oriented contributed to alienation among some. Collecting data on infection rates often did not always produce a norm-disrupting effect that challenged units to change their practices and cultures; instead, in some units, it reinforced the status quo. Features of ICUs’ internal context also appeared relevant to explaining how they responded to the program. In units where change—either transformative or boosting—was attributed by staff to *Matching Michigan*, several things needed to happen. At least one senior physician needed to exert strong leadership, and to work in coalition with senior nurses. These leaders needed to be recognized by their peers as committed, credible, and engaging, and to communicate a sincere belief in the program. They needed to do enough listening to ensure peers felt heard, but also know when to ‘push it hard.’ Data collection systems needed to be rigorous enough to command credibility. Leaders also needed to be canny enough to appropriate the centrally-led, external nature of the program to confer authority and legitimacy on their efforts and to drive through improvements that would otherwise have been difficult to achieve.

The broader context in which *Matching Michigan* was launched was highly influential in modifying its possibilities for effecting change, and further helps in explaining the possible decline effect. The Keystone project had the benefit of newness and freshness; ICUs in England, on the other hand, had been exposed to a long series of initiatives and pressures in relation to infection control, and believed that they had already made changes in response.

The possibility that staff in English ICUs would, as was theorized to have occurred in Keystone, come to recognize CVC-BSIs as a social problem capable of being solved [10] was undermined by their perception that it was a problem that had already been solved. *Matching Michigan* was seen by some staff as a failure to respect what they had already achieved, and, given the other challenges facing hospitals, a misdirection of resources. The goals, interests and priorities of the program were therefore widely seen as misaligned with those of staff at the sharp-end. Further, it may well have been the case that in some units there genuinely was very little headroom for improvement: all the gains that could be made had already been made, and in such contexts improvement programs characteristically struggle to demonstrate further change [14,22].

Importantly, our study does not challenge the efficacy of the *Matching Michigan* interventions (data collection, non-technical and technical) in improving compliance with evidence-based practices and reducing infection rates. Much depended on context: program interventions were able to deliver positive change where they were implemented. As the ‘transformed’ unit showed, under the right conditions and deployed appropriately, these interventions could be used to bring about substantial improvements. The disappointment of the program is that it did not achieve these effects on a larger scale; some ICUs that could have benefited from strengthening their organizational culture and consistency of good practice did not take advantage of the opportunity to do so. We propose that though the interventions ‘work,’ *Matching Michigan* did not fully work as program because of features of program design and delivery and contexts of implementation.

None of this is to deny that improvement in rates of CVC-BSIs did occur while *Matching Michigan* was running. Almost two-thirds of units across the program reported no infections, meaning a median of zero—the same as seen in Keystone. A 60% decline in infection rates over the course of the program is non-trivial, even if it is difficult to pin the credit on the program.

Only one other study appears to have concurrently studied CVC-BSIs acquired outside the ICU, and this too identified a strong secular trend [23] suggesting that ‘rising tides’ could have contributed to the apparent success of other studies in this area. Our study offers some insights into the causes of the rising tide and why it was hard to detect a program effect in excess of the secular trends. *Matching Michigan* came into being because there was already strong policy and professional pressure—not least because of the success of Keystone—to do better in reducing rates CVC-BSIs. A recently published analysis of 20 years of data shows that CVC-BSI rates began to plummet from 1990 onward, and the increasing rarity of the infections is likely to have undermined the view that they were the price of doing business in ICUs [24,25]. The forces that brought the program into being continued to intensify over the course of the program. All ICUs in England were all part of an institutional ecology exposed to the same environmental pressures that caused them, over time, to become increasingly sensitized to expectations of good practice. Each initiative, program, and statement of professional and scientific consensus is likely to have played a cumulative and mutually reinforcing role, though none that could easily be discerned individually. A similar effect is often seen in community health promotion programs [26]. The main function of programs such as *Matching Michigan* in a context where there is an overall trend in a particular direction may be to add to the pressures and provide structures and legitimacy for change. Had the relevance of the context been more fully acknowledged, and the program more faithful to the hypothesized mechanisms of the original, more might have been achieved.

Our study has a number of important limitations. Our ethnographic visits to units were not longitudinal, but rather snapshots in time; changes in response to the program could have occurred after our visits. We did not conduct a systematic audit of culture and practices, and thus some inaccuracies in our assessments may be present. We did not evaluate possible modifiers of effect of factors such as size of unit, number of consultants and nurses, and other environmental features. We had access to ICUs’ reported infection rates only if they provided them directly to us; for information governance reasons, these rates could not be verified. It is possible that we have offered too pessimistic an interpretation of whether *Matching Michigan* ‘worked’: the quantitative evaluation [13] may have underestimated the effects of the program (or over-estimated the secular trend), since the ‘waiting’ clusters were not true controls that were unexposed to the interventions. It is also possible that our comparison with the original Keystone program may be flawed in the direction of optimism: it is possible that it did not function in the way theorized. There is no way of verifying its mechanisms, because contemporaneous ethnographic data were not collected, and the study design lacked controls.

## Conclusions

Our study points to the need for clear understanding of the mechanisms by which improvement programs work. Though *Matching Michigan* reproduced many of the components of the original Keystone project, it did not reproduce many of the features of Keystone that were theorized to have enabled it to work. This, together with the contexts in which it was introduced, helps to explain both the decline effect and the apparent failure to outperform the secular trend. Our analysis will inform future research in this area [27] by demonstrating the importance of distinguishing technical interventions from implementation strategies, and the need for careful attention to the contexts in which improvement programs are introduced if they are to deliver maximum benefit for patients.

### Ethical approval

A favorable opinion was obtained from Berkshire NHS Research Ethics Committee was obtained for this study.

## Competing interests

Julian Bion held the advisory role of senior clinical lead for the *Matching Michigan* project, conducted in parallel with the ‘Lining Up’ project on which this article is based. His employers were financially compensated for his time by the National Patient Safety Agency. No other authors declare financial relationships with any organizations that might have an interest in the submitted work in the previous three years or other relationships or activities that could appear to have influenced the submitted work No other authors have a conflict of interest.

## Authors’ contributions

MDW, CT, and JB conceived of and designed the study, conducted the literature review, obtained the funding, and recruited participating sites. MDW and CT led on obtaining ethics and governance approvals. CT and ML undertook ethnographic observations and interviews in participating sites. MDW and CT coordinated the study. MDW, CT, and ML devised the analytic framework. ML undertook coding, which was checked by MDW. MDW and ML led on drafting of the manuscript, with extensive contributions from CT and JB. All authors read and approved the final manuscript.

## Access to data

MDW, CT, and ML had full access to all data and can take responsibility for the data and accuracy of data analysis. For ethical reasons it was not possible to share all raw data with JB. He did not have access to any primary data from ‘Lining Up’ while *Matching Michigan* was running, and analysis of the ‘Lining Up’ data was conducted independently of him.
